# Evidence of low prevalence of mycobacterial lymphadenitis in wild boars (*Sus scrofa*) in Poland

**DOI:** 10.1186/s13028-017-0277-0

**Published:** 2017-01-25

**Authors:** Lucjan Witkowski, Blanka Orłowska, Magdalena Rzewuska, Michał Czopowicz, Mirosław Welz, Krzysztof Anusz, Jerzy Kita

**Affiliations:** 1 Laboratory of Veterinary Epidemiology and Economics, Faculty of Veterinary Medicine, Warsaw University of Life Sciences-SGGW, Nowoursynowska 159c, 02-776 Warsaw, Poland; 2 Department of Food Hygiene and Public Health Protection, Faculty of Veterinary Medicine, Warsaw University of Life Sciences-SGGW, Nowoursynowska 159, 02-776 Warsaw, Poland; 3 Department of Preclinical Sciences, Faculty of Veterinary Medicine, Warsaw University of Life Sciences-SGGW, Ciszewskiego 8, 02-786 Warsaw, Poland; 4 Voivodeship Veterinary Inspectorate in Krosno, ks. Piotra Ściegiennego 6 A, 38-400 Krosno, Poland

**Keywords:** *Mycobacterium* spp., Abscess, Wildlife

## Abstract

*Mycobacterium* spp. and *Rhodococcus equi* are generally regarded as the main causes of lymphadenitis in pigs and wild boars. In Poland, mycobacterial submandibular lymphadenitis was first diagnosed in a wild boar in 2012 but *Mycobacterium* spp. infections are also present in the Polish population of European bison (*Bison bonasus*). The prevalence of lymphadenitis in Polish wild boars has been found to 8.4% (95% CI 6.2–11.3%) and it has been proved that *R. equi* is not an important cause of purulent lesions in these animals. The current study was carried out to assess the prevalence of mycobacterial lymphadenitis in the Polish wild boar population. Submandibular lymph nodes with purulent lesions collected from 38 wild boars in 2010/2011 and negative for *R. equi* were included. Calculations based on the hypergeometric approximation were used to determine the probability that at least one positive individual would be detected if the infection had been present at a prevalence greater than or equal to the design prevalence. All 38 samples were negative for *Mycobacterium* spp. [0% (95% CI 0, 9.2%)]. Epidemiological analysis showed that the true prevalence was 95% likely to be lower than 10%. In conclusion, mycobacterial lymphadenitis seems to occur rarely in wild boars in Poland. Due to the presence of *Mycobacterium* spp. infections in other wildlife, the surveillance of mycobacterial infections in wild animals in Poland remains an important issue.

## Findings

Wild animals play an important role in the epidemiology of infectious diseases as reservoirs of several zoonotic and non-zoonotic diseases. Tuberculosis (TB) is one of the most important diseases affecting wild and domestic animals and also humans [1]. TB in wild boars and feral pigs is a growing problem in some European countries. These animals are much more sensitive TB-sentinels than other wildlife species and are considered to be not only a spill-over but also reservoir hosts or even super-shedders excreting significantly higher amounts of *Mycobacterium* spp. bacteria than standard shedders [2].

In Europe, the prevalence of TB in wild boars differs among countries and even within regions [3–8]. Tuberculosis in wild boars has been reported in several European countries such as Spain [3], Italy [4], Portugal [7], Great Britain [5], France [6] and recently in Poland [8].

Tubercular lesions in wild boars are typically caseocalcareus. They consists of tubercles with diameters up to 5 cm with a dry yellow content or greenish pus or as 1 mm sized miliary foci, located mostly in the lymph nodes of the head, usually the submandibular lymph nodes [3, 6].

Tuberculosis caused by *Mycobacterium bovis* or *M. caprae* in wild boars have been reported most frequently [2, 3, 5–8], while *M. microti* [4] and non-tuberculous, potentially pathogenic environmental mycobacteria, have been reported less often [9, 10].

The diagnosis of *Mycobacterium* spp. infection in free-ranging wildlife is relatively difficult and relies on post-mortem examination. Laboratory diagnosis is based mainly on microscopic examination of Ziehl-Neelsen stained slides and bacterial cultivation. Histopathology may be ambiguous as lesions caused by various mycobacterial species are difficult to distinguish. Different targeted polymerase chain reaction (PCR) assays as “*IS6110*” sequence are useful and reliable for the detection of mycobacteria in clinical specimens [11]. However, their sensitivity varies and may be low [12]. Therefore, culture is considered the gold standard [13, 14] due to the highest specificity of all available methods. It may however produce false-negative results and its sensitivity has been estimated at approximately 80% [7].

**Fig. 1:**
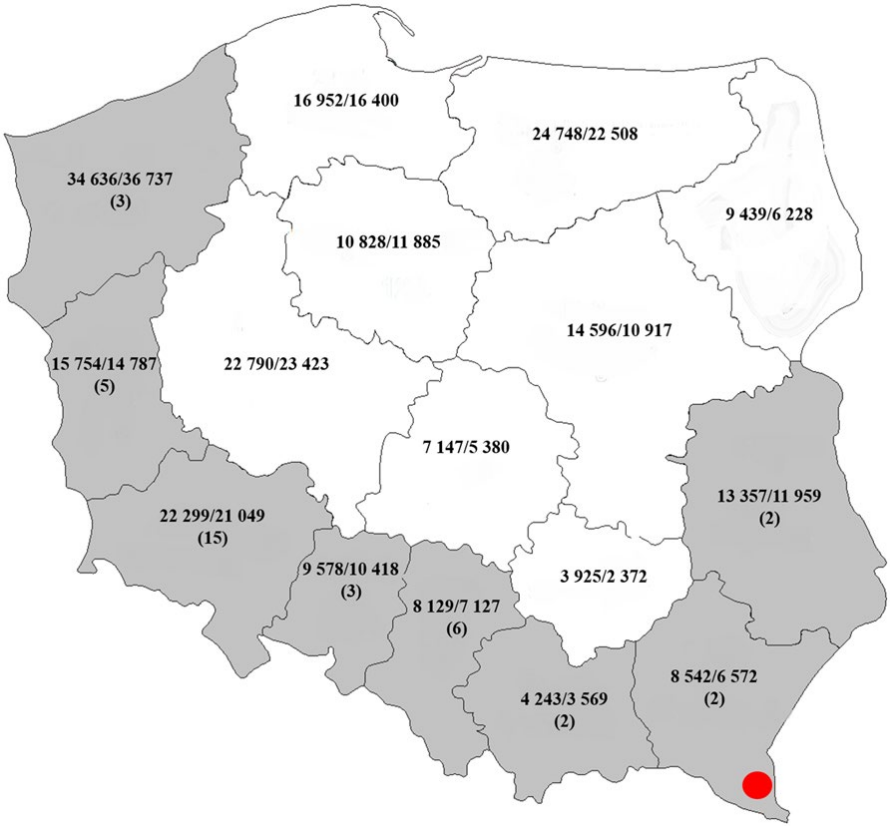
Population of wild boar and the number of hunted animals in Poland in the 2010/2011 season (data of the Polish Hunting Association) and origin of investigated samples. Figures on the map denote No. of adult wild boars/no. of all, adult and young hunted wild boars. Voivodeships from which the submaxillary lymph nodes with purulent lesions were collected are shaded. No. of tested samples are in parentheses. The area where TB in bison and wild boar has occurred since 1996 is marked by *red dot*

In Poland, mycobacteriosis is an emerging disease of wildlife, and was recognized for the first time in the European bison (*Bison bonasus*) in the Bieszczady Region in 1996 (Fig. 1) and has since then become an increasing problem [15]. Moreover, in 2012, *M. bovis* was isolated from submandibular lymph node lesions of a wild boar in that region [8].

Tuberculous-like lesions in lymph nodes in livestock and wild animals can be caused not only by *Mycobacterium* spp. and *R. equi* but also by other aerobic and anaerobic bacteria including *Staphylococcus* spp., *Streptococcus* spp., *Corynebacterium* spp. or *Trueperella pyogenes* [9, 16–18]. In the last decade, *R. equi* has raised considerable interest because of its zoonotic potential and the similarity to tubercular lesions. In domestic pigs, *R. equi* has been recognized as the main cause of lymphadenitis [17] but it has also been isolated from lymphadenitis in wild boars in Brazil [9, 10] and from purulent lesions in American bison (*Bison bison*) co-infected with *Mycobacterium* spp. [19]. On the other hand, *R. equi* did not prove an important cause of lymphadenitis in Polish wild boars [20] but was isolated from the lymph nodes of apparently healthy wild boars intended for human consumption [20]. According to the data of the Polish Hunting Association in the 2010/2011 season, the population of adult wild boars in Poland was estimated at 226,936 heads while 211,331 animals (both young and adult) were hunted.

In this study, inflamed submandibular lymph nodes from 38 wild boars hunted in the 2010/2011 season (Fig. 1) were analyzed. These samples have previously been used in another study [20]. All samples were negative for *R. equi* and most of the lesions were apparently indistinguishable from typical *Mycobacterium*-associated lesions. The samples were stored at -20 °C for approx. 24 months. A standard procedure according to the Manual of the World Organization for Animal Health (OIE) was used for the identification of *Mycobacterium* spp. Briefly, the thawed tissue samples were soaked and homogenized in 5% oxalic acid. The suspension was incubated at 37 °C for 10–15 min and centrifuged at 11 000×*g* for 10 min. The pellets were washed with 0.9% saline and inoculated onto Stonenbrink’s and Loewenstein–Jensen’s media supplemented with glycerin and pyruvate respectively (Oxoid, Postfach, Germany). The samples were incubated at 37 °C for 12 weeks with weekly readings. Media containing *M. caprae* and *M. avium* were used as positive controls. Mycobacteria were identified on the basis of colony growth and morphology according to [15]. Additionally, the part of thawed tissue samples were cultured on Columbia Agar supplemented with 5% sheep blood (bioMerieux, Grenoble, France) and incubated at 37 °C in microaerophilic conditions.

Given that lymphadenitis, regardless of its cause, was found in 6–11% of Polish wild boars [20] and the general population of wild boars in Poland consists of approximately 200,000 adults, the population of lymphadenitis-affected wild boars was estimated at 20,000 animals. For the needs of epidemiological analysis, culture sensitivity and specificity were assumed to be 80% and 100%, respectively [7, 13]. Calculations based on the hypergeometric approximation were used to determine the probability (level of confidence of population freedom, LoC) that at least one positive individual would have been detected if the disease had been present at a prevalence greater or equal to the design prevalence. The following formula was used: LoC = 1 − (1 − TSe × n/N)^DP^ where n denotes a sample size, N—population size, DP—design prevalence and TSe—test sensitivity of 80% [7].

The design prevalence included in the study ranged from 1 to 20%. The epidemiological analysis was performed in EpiTools [21]. A 95% confidence interval (95% CI) for prevalence was calculated using Wilson score method [22].

**Fig. 2:**
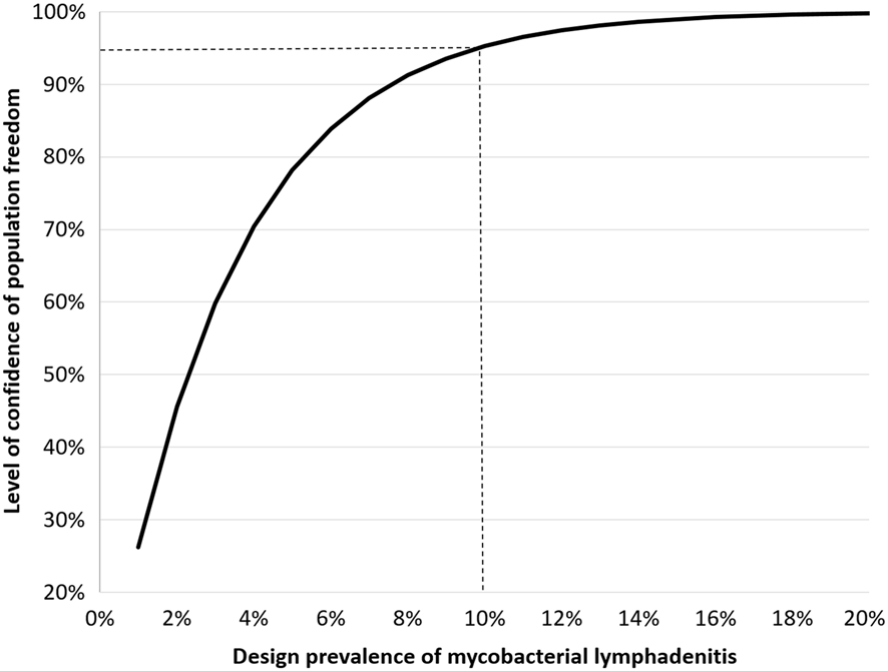
Probability (level of confidence of population freedom) that the mycobacterial lymphadenitis prevalence in wild boars in Poland is lower than the design prevalence. Broken lines indicate design prevalence corresponding to a 95% level of confidence

All investigated samples (n = 38) tested negative for *Mycobacterium* spp. yielding a true prevalence of *Mycobacterium* spp. infection in lymphadenitis-affected wild boars of 0% (95% CI 0, 9.2%). Epidemiological analysis showed that the true prevalence was 95% likely to be lower than 10% (Fig. 2). No other pathogenic bacteria such as *Corynebacterium* spp. or *T. pyogenes* were detected. Only nonpathogenic environmental bacteria such as *Bacillus* spp., *Flavobacterium* spp. and *Micrococcus* spp. were cultivated.

Data regarding different pathogens isolation from wild boar lymph nodes with purulent lesions are scarce and limited to the two reports from Brazil [9, 10] in which *Mycobacterium* spp. were isolated in 8.4% of the cases, *R. equi* in 6.6%, *T. pyogenes* in 5.4%, and *Staphylococcus* sp., *Streptococcus* sp. and other bacteria in 2–3%. Interestingly, 18.6% of investigated samples were negative for any bacteria as were all samples investigated in this study. However, contrary to Europe, wild boars in Brazil are not wildlife, but exotic for the local fauna, kept on commercial farms in semi-extensive conditions and the results should be compared with caution.

This study has several limitations. Freezing of the tissues precluded histopathological examination and the long storage time at −20 °C could potentially have influenced the viability of the bacteria although it has been shown that the time of storage at −20 °C had no significant effect on the rate of *M. tuberculosis* recovery [23]. In this study, samples of purulent lesions typical for *Mycobacterium* spp. infection were investigated and a high number of culture positive samples was expected. Influence of sample storage of other bacterial pathogens survival could not be excluded. PCR was not performed because the samples, which were left from a previous project, were intended for culture.

Differences in the prevalence of *Mycobacterium* spp. infection in various wild boar populations in other countries may have several explanations. One can be different prevalence of other infections positively linked to TB severity in wild boar [3] such as infections with porcine circovirus type 2, Aujeszky’s disease virus and *Metastrongylus* spp., which all are also present in wild boar population in Poland [24]. The different prevalences can also be influenced by environmental factors such as high density of wildlife, contact with livestock or presence of the known TB-reservoir species [25]. In Poland wild boar population is growing (from 120,000 in 1999/2000 to 285,000 in 2014/2015) and contact with livestock is possible. So far TB in wild animals in Poland has been restricted to the Bieszczady Region and other TB-reservoir species than the European bison population remain unknown [8, 15]. Poland is officially free of bovine TB since 2009 (Commission Decision 2009/342/EC).

We conclude that in the 2010/2011 hunting season, mycobacterial lymphadenitis in wild boars was less prevalent in Poland than in other European countries. Due to the presence of *Mycobacterium* spp. infection in the European Bison population in the Bieszczady Region, a growing wild boar population, and the presence of pathogens predisposing wild boars to TB, the surveillance of mycobacterial infections in this species is necessary.

## Abbreviations

95% CI: 95% confidence interval
TB: tuberculosis
PCR: polymerase chain reaction
